# Editorial: Novel strategies for the clinical management of cardiovascular-kidney-metabolic syndrome

**DOI:** 10.3389/fendo.2026.1818442

**Published:** 2026-03-09

**Authors:** Yun Shen, Junfeng Han, Xiaoying Ding

**Affiliations:** 1 Division of Population and Public Health Science, Pennington Biomedical Research Center, Baton Rouge, LA, United States; 2 Department of Endocrinology and Metabolism, Tongji Hospital, School of Medicine, Tongji University, Shanghai, China; 3 Department of Endocrinology and Metabolism, Shanghai General Hospital, Shanghai Jiaotong University School of Medicine, Shanghai, China

**Keywords:** cardiovascular kidney metabolic (CKM) syndrome, clinical trial, epidemiology, population studies, real world evidence (RWE)

The cardiovascular–kidney–metabolic (CKM) syndrome framework formalizes what clinicians have long observed: obesity, dysglycaemia, chronic kidney disease (CKD), and cardiovascular disease (CVD) are not parallel epidemics but intertwined disorders connected by shared pathobiology (insulin resistance, glucolipotoxicity, endothelial dysfunction, inflammation, oxidative stress, and maladaptive neurohormonal activation) that accelerate organ damage and premature death. The studies assembled in this Research Topic span population epidemiology, risk prediction, staging-informed prognosis, and therapeutic strategies—together offering a pragmatic roadmap for moving CKM from a conceptual model to actionable clinical pathways.

## From staging to prognosis: quantifying risk across the CKM continuum

Several contributions leverage the CKM staging construct to sharpen prognostication and identify when interventions may yield the greatest health gains. In a large Chinese cohort, Dai et al. examined advanced CKM and showed that age at onset meaningfully reshapes both relative and absolute mortality burdens, supporting an age-stratified prevention and management strategy with distinct “windows” for efficient risk interception and later burden reduction. This life-course framing complements Meng et al. CKD-focused cohort study, where higher CKM stages were associated with greater risks of all-cause mortality and progression to end-stage renal disease, underscoring that CKM staging can meaningfully stratify outcomes even within already high-risk CKD populations.

Two additional studies highlight the growing role of integrated inflammatory–metabolic markers as “bridges” linking CKM stage to outcomes. Han et al. reported that the hs-CRP/HDL-C ratio predicted long-term mortality among individuals with CKM stages 1–4, and that adding this ratio improved risk prediction beyond baseline factors. In parallel, Zhang et al. evaluated the atherogenic index of plasma (AIP) in CKM stages 1–3, emphasizing cumulative exposure and “control patterns” as determinants of subsequent CVD risk—an approach consistent with the idea that CKM progression is driven by long-term metabolic injury rather than single-time-point biomarkers (1). Finally, Jiang et al. demonstrated that semi-quantitative ultrasound assessment of carotid and lower extremity atherosclerosis correlates with coronary lesion severity and may serve as a practical, noninvasive adjunct for predicting the need for coronary revascularization.

## Refining anthropometric and metabolic risk markers: beyond BMI, toward actionable phenotypes

A major theme across the Research Topic is the need to improve early identification of high-risk CKM phenotypes. Zhou et al. used NHANES data to compare multiple “novel adiposity indices” with BMI for mortality prediction among individuals with CKM stages 0–3, reinforcing that body fat distribution and related indices may better capture risk than BMI alone in early CKM. Complementing this, Xu et al. evaluated TyG-BMI in a large clinical cohort with type 2 diabetes and linked it to a broad panel of cardiovascular, renal, hepatic, and bone biomarkers—supporting insulin-resistance–anchored composite indices as practical tools for mapping multisystem vulnerability in diabetes-related CKM.

The importance of phenotype-level stratification is also underscored by Chen et al. who applied latent class analysis in patients with type 2 diabetes and CKD and identified metabolic profiles that predicted renal composite outcomes over time. Rather than treating “metabolic syndrome” as a binary diagnosis, their work illustrates a precision-nephrology direction: using multidimensional metabolic patterns to identify those most likely to benefit from intensified risk-factor modification and renal-protective therapies.

## CKM in special populations: multimorbidity signals and survivorship care

The Research Topic also extends CKM beyond conventional cardiometabolic cohorts. Zou et al. investigated osteoarthritis (OA) as a potential clinical marker of CKM multimorbidity and showed higher OA burden was associated with increased odds of developing single, double, and triple CKM outcomes, supported by multi-state modelling of disease transitions. These findings align with a growing view that OA can represent systemic metabolic–inflammatory dysregulation rather than an isolated joint disorder, and they prompt consideration of musculoskeletal clinics as opportunistic settings for CKM screening.

Similarly, Liu et al. focused on colorectal cancer survivorship and showed that baseline metabolic syndrome was associated with higher incident CVD risk among cancer survivors, reinforcing that CKM-oriented risk factor assessment and prevention should be embedded in survivorship models rather than deferred to general primary care. Together, these studies emphasize that CKM is not simply a cardiology/endocrinology construct; it is a cross-disciplinary care challenge that appears across clinical pathways where prevention infrastructure is often weakest.

## Therapeutic strategies: renal–cardiometabolic protection as the treatment backbone

The Research Topic includes both pharmacologic and care-delivery–oriented strategies to mitigate CKM progression. The review by Wang et al. synthesizes mechanistic and clinical evidence supporting sodium–glucose cotransporter 2 inhibitors (SGLT2i) as a cornerstone therapy across CKM domains, highlighting glucose-lowering–independent benefits (haemodynamic effects, anti-inflammatory/anti-fibrotic actions, renal protection, and cardiovascular event reduction).

In addition, Li et al. reported results from a Chinese subgroup analysis of FIGARO-DKD, supporting the cardiovascular and kidney benefits of finerenone in patients with CKD and type 2 diabetes on optimized renin–angiotensin system blockade—an important step toward contextualizing trial evidence in real-world Chinese populations. These pharmacologic contributions reinforce an emerging consensus: CKM management should prioritize therapies with multi-organ benefit, especially in populations with overlapping CKD and diabetes where single-organ approaches are clinically inefficient.

Three further studies provide real-world and risk-factor insights that can guide clinical implementation. Gong et al. evaluated short-term comprehensive treatment in type 2 diabetes and reported improvements in CKM staging and reductions in 10-year CVD risk scores after intensive management. Zhang et al. examined early-onset type 2 diabetes and found that advanced CKM burden increases sharply with diabetes duration, identifying renal markers (eg, albuminuria) and disease duration as key correlates—highlighting the need for early kidney-focused prevention in early-onset diabetes. Finally, Wang et al. examined post–COVID-19 type 2 diabetes and assessed predictors of worsening 10-year CVD risk, reflecting a timely clinical question: how acute systemic stressors may interact with chronic metabolic vulnerability to accelerate cardiovascular risk trajectories.

## What this Research Topic collectively suggests for practice and policy

Across heterogeneous settings, a coherent message emerges: CKM care should be staged, phenotype-informed, and intervention-timed. First, CKM staging can operationalize risk communication and triage—particularly when enriched with markers of inflammation and dyslipidaemia that appear to refine mortality prediction. Second, better phenotyping of adiposity and insulin resistance—using indices beyond BMI and single biomarkers—may enable earlier identification of high-risk patients before irreversible kidney and cardiovascular complications develop. Third, therapies that protect multiple organs (SGLT2i, mineralocorticoid receptor antagonism with finerenone, and comprehensive risk-factor programs) should form the backbone of pragmatic CKM pathways, supported by multidisciplinary delivery models that extend into oncology survivorship and musculoskeletal care.

From a health-systems and policy standpoint, these papers also highlight practical implementation opportunities: (1) embed CKM screening in high-throughput clinics (diabetes, nephrology, oncology survivorship, orthopedics); (2) standardize a minimal set of scalable measures (waist-based adiposity indices, albuminuria, eGFR, inflammation/lipid composite markers) for staging-informed risk stratification; and (3) design age- and sex-responsive pathways that allocate resources to periods of maximal preventability.

## Future directions

Despite important advances, the Research Topic also points to key next steps: prospective validation of novel indices across diverse ethnicities and care settings; causal modelling to distinguish risk markers from modifiable mediators; pragmatic trials testing staged, multidisciplinary CKM care pathways; and health-economic evaluations of integrated pharmacotherapy (eg, SGLT2i + finerenone where indicated) combined with structured lifestyle and risk-factor management.

In conclusion, these 14 papers collectively move the field toward a more operational CKM paradigm—one that is measurable, stratifiable, and increasingly treatable through therapies and care models designed for multi-organ benefit.

## References

[1] Zhang Y Song Y Lu Y Liu T Yin P . Atherogenic index of plasma and cardiovascular disease risk in cardiovascular-kidney-metabolic syndrome stage 1 to 3: a longitudinal study. Front Endocrinol (Lausanne). (2025) 16:1517658. doi: 10.3389/fendo.2025.1517658, PMID: 39968297 PMC11832398

